# Mice with endothelial cell‐selective adhesion molecule deficiency develop coronary microvascular rarefaction and left ventricle diastolic dysfunction

**DOI:** 10.14814/phy2.15643

**Published:** 2023-03-22

**Authors:** Vadym Buncha, Katie Anne Fopiano, Liwei Lang, Celestine Williams, Anatolij Horuzsko, Jessica Andrea Filosa, Gaston Kapuku, Zsolt Bagi

**Affiliations:** ^1^ Department of Physiology Medical College of Georgia, Augusta University Georgia Augusta USA; ^2^ Department of Medicine Georgia Prevention Institute, Medical College of Georgia, Augusta University Augusta Georgia USA; ^3^ Georgia Cancer Center Medical College of Georgia, Augusta University Georgia Augusta USA

**Keywords:** angiogenesis, coronary, diastolic dysfunction, HFpEF, inflammation

## Abstract

Endothelial cell‐selective adhesion molecule (ESAM) regulates inflammatory cell adhesion and transmigration and promotes angiogenesis. Here, we examined the role of ESAM in cardiac vascularization, inflammatory cell infiltration, and left ventricle (LV) diastolic function under basal and hemodynamic stress conditions. We employed mice with homozygous genetic deletion of ESAM (ESAM^−/−^) and also performed uninephrectomy and aldosterone infusion (UNX‐Aldo) to induce volume and pressure overload. Using echocardiography, we found that ESAM^−/−^ mice display no change in systolic function. However, they develop LV diastolic dysfunction, as indicated by a significantly reduced E/A ratio (E = early, A = late mitral inflow peak velocities), increased E/e’ ratio, isovolumic relaxation time (IVRT), and E wave deceleration time. An unbiased automated tracing and 3D reconstruction of coronary vasculature revealed that ESAM^−/−^ mice had reduced coronary vascular density. Arteries of ESAM^−/−^ mice exhibited impaired endothelial sprouting and in cultured endothelial cells siRNA‐mediated ESAM knockdown reduced tube formation. Changes in ESAM^−/−^ mice were accompanied by elevated myocardial inflammatory cytokine and myeloperoxidase‐positive neutrophil levels. Furthermore, UNX‐Aldo procedure in wild type mice induced LV diastolic dysfunction, which was accompanied by significantly increased serum ESAM levels. When compared to wild types, ESAM^−/−^ mice with UNX‐Aldo displayed worsening of LV diastolic function, as indicated by increased IVRT and pulmonary edema. Thus, we propose that ESAM plays a mechanistic role in proper myocardial vascularization and the maintenance of LV diastolic function under basal and hemodynamic stress conditions.

## Graphical abstract

Endothelial cell‐selective adhesion molecule (ESAM) regulates inflammatory cell adhesion and transmigration and promotes angiogenesis. We propose that animals deficient in ESAM develop left ventricular diastolic dysfunction due to reduced myocardial vascular density, hypoxia, and inflammation. Hemodynamic challenge lead to worsening of left ventricular diastolic dysfunction in ESAM knockout mice.

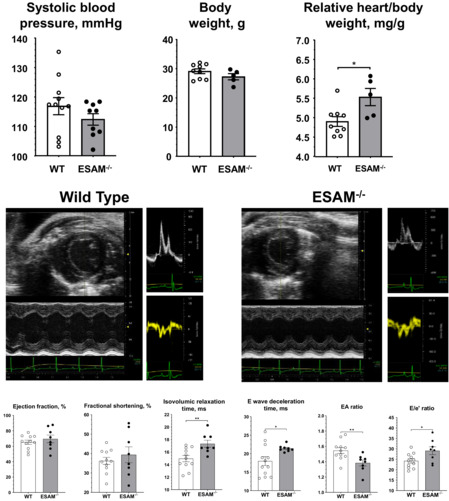

## 1 INTRODUCTION

The endothelial cell‐selective adhesion molecule (ESAM) is an immunoglobulin‐like cell adhesion molecule and a member of the family of endothelial cell junctional adhesion molecules (Hirata et al., 2001). ESAMs unique position at tight endothelial junctions favors its homophilic arrangement and molecular interactions on adjacent endothelial cells (Weber et al., 2007). Functionally, homophilic interactions of ESAM stabilize endothelial cell junctions, hence controlling cell permeability, and are also important determinants of inflammatory cell transmigration (Bazzoni, 2006; Liu et al., 2000; Mandell et al., 2004; Martin‐Padura et al., 1998; Wegmann et al., 2006). Interestingly, ESAM was also shown to play a role in the angiogenic process (Inoue et al., 2010). Studies have found that in mice with genetic ESAM deletion, tumor vascularization and tumor growth are reduced (Ishida et al., 2003). ESAM deficiency also delayed atherosclerotic lesion formation by inhibiting plaque neovascularization in mice (Inoue et al., 2010).

The tissue expression of ESAM appears to be organ‐specific in that ESAM is abundantly expressed in the heart and lung with lower expression levels detected in other organs, such as the kidney and skin (Hirata et al., 2001). In addition, the extracellular domain of ESAM can be cleaved, by an unidentified mechanism, and secreted into the bloodstream in a soluble form. Of clinical importance, elevated serum levels of ESAM have been evaluated in cardiovascular diseases (Ren et al., 2017). A prior study reported a close association between elevated ESAM levels in serum and the incidence of myocardial infarction, heart failure hospitalizations, and death, and found that the positive correlation remains significant after adjusting for demographic factors and clinical parameters, such as NT‐proBNP levels (Park et al., 2015). Another study observed a positive correlation between serum ESAM and albuminuria in patients with coronary artery disease (Park et al., 2014).

The mechanistic interrelationships between ESAM and cardiovascular functioning, including the role of ESAM in myocardial vascularization, inflammatory cell infiltration, and cardiac pump function, as well as the potential clinical importance of ESAM during cardiovascular pathologies remain elusive. Therefore, in this study we employed mice with homozygous genetic deletion of ESAM (ESAM^−/−^) and specifically assessed myocardial vascularization, inflammatory cell infiltration, and left ventricle (LV) diastolic function both under basal and hemodynamic stress conditions.

## 2 METHODS

### 2.1 Animals

The work involving experimental animals was conducted under the protocol approved by the Institutional Animal Care and Use Committee at Medical College of Georgia, Augusta University. Experiments were carried out in 16–20 weeks old male ESAM^−/−^ and C57BL/6J wild type littermates (Strain #:005862, Jackson Laboratory). The mice were housed in the animal care facility and accessed rodent chow and tap water ad libitum with a 12‐h light:dark cycle.

### 2.2 Echocardiography

A high frequency ultrasound system (VEVO 3100, VisualSonics, Toronto, ON, Canada) was used to assess cardiac function in anesthetized mice (2% isoflurane). M‐mode was used in parasternal short‐axis for systolic function estimation, while pulsed‐wave Doppler and tissue Doppler were utilized to estimate diastolic function (pressure gradients and blood flow parameters across the mitral valve) in the four chamber apical view (Lindsey et al., 2018; Respress & Wehrens, 2010).

### 2.3 Blood pressure

Tail‐cuff plethysmography method (CODA, Kent Scientific, Torrington, CT) was utilized to measure blood pressure in conscious mice after 5 days habituation. Duration of the measurement session was ≤30 min for which animals were placed on a heated (36°C) platform.

### 2.4 Quantification of coronary microvascular density

Heart samples were fixed in 4% paraformaldehyde and embedded in paraffin. Sections were cut (40 μm) and immunolabeled with Tomato‐Lectin DyLight 594 Antibody (1:150, overnight, 4°C; DL‐1177, Vector Laboratories) for the visualization of vascular networks. Z‐stacks (h × l × w = 25 × 350 × 500 μm, single scan thickness 0.5 μm) were taken randomly from 3–4 different locations with structured illumination microscope (SIM‐Apotome, AxioImagerM2, CarlZeiss). Spatial reconstruction and morphological analysis of the cardiac microvasculature was performed with the Vesselucida 360 software (v2018.1.1, MBF Bioscience) using data obtained from the Z‐stacks. Automatic reconstruction of the vasculature from all samples was performed utilizing the Rayburst Crawl algorithm, tracing with seed sensitivity 80, medium seed density with refine filter 2, and medium gap tolerance. Total vascular length, total surface area, and volume of the vasculature were determined. Average diameter of the vessel was calculated. For the final analysis, structures with a large diameter and small length (D/L > 2) were excluded as algorithm‐generated artifacts.

### 2.5 Ex vivo artery ring angiogenic assay

For the ex vivo ring angiogenic assay, the carotid artery was dissected from ESAM^−/−^ and wild type mice immediately after animal sacrifice and placed in 1X PBS. Vascular rings were cleared from perivascular adipocytes and connective tissue. Cut in equal length (~5 mm), rings were embedded into a collagen matrix mixture containing Collagen Type I (354,236, Corning), DMEM (10‐013‐CV, Corning), and 1 M NaOH (BP359‐500, ThermoFisher) in a 96‐well plate and placed in an incubator at 37°C, 5% CO_2_. After 6 h of incubation, 150 μL of growth media containing Opti‐MEM® (11058–01, Gibco), Fetal Bovine Serum (10437–028, Gibco), Penicillin–Streptomycin (15,070,063, Gibco), GlutaMAX™ (35050–061, Gibco), and recombinant VEGF (493‐MV‐025/CF, R&D Systems) was added to each 96‐well. Following Day 0, growth media was exchanged for fresh media on Day 3 and Day 5. Endothelial sprouting was assessed via a brightfield microscopy and sprout number per vessel ring on days 3, 5, and 7 were counted.

### 2.6 Endothelial tube formation assay

Human umbilical vein endothelial cells (HUVECs) were purchased from PromoCell (Heidelberg, Germany). HUVECs were cultured in Endothelial Cell Growth Medium 2 and used between 4–10 passages. HUVECs (10^5^ cells/slide) were seeded onto IbiTreat μ‐slides I^0.6^ (Ibidi) and placed in an incubator at 37 °C with 5.0% CO_2_. Transfection of HUVECs with ESAM small interfering RNA (siRNA, Dharmacon™) was carried out using Lipofectamine® RNAiMAX Transfection Reagent (ThermoFisher Scientific). Tube formation was quantified by automated measurements of total tube length, branching nodes, number of nodes, and branching intervals using ImageJ.

### 2.7 Immunohistochemistry

Heart samples were fixed in 4% paraformaldehyde (sc‐281,692, Santa Cruz) and were embedded in paraffin. Sections were cut (8 μm) and re‐hydrated through sequential incubations in a series of graded xylene (X3P‐1GAL, Fisher Scientific), ethanol (BP2818, Fisher Scientific), and phosphate buffer solution (PBS). Antigen retrieval was performed by running slides submerged in IHC‐TekTM Epitope Retrieval Solution. Slides were blocked with 15% horse serum (S‐2000, Vector Laboratories) for 1 h and followed by incubation with anti‐MPO antibody (1:400, ab208670, Abcam). DAB labeling was performed with HRP‐conjugated donkey anti‐rabbit IgG (1:250, A10040, Invitrogen). Hematoxylin (H‐3404, Vector Laboratories) was used for nuclear staining. For the result quantification, 10 pictures were taken from each sample and the average number of cells per picture was calculated.

### 2.8 Uninephrectomy, aldosterone infusion, and high salt treatment in mice

ESAM^−/−^ and wild type mice underwent an established surgical procedure to induce experimental pressure and volume overload, as described previously (Valero‐Muñoz et al., 2016). In brief, we performed uninephrectomy together with vehicle (UNX) or aldosterone (Aldo; 15,273, Cayman Chemical) infusion (2004, Alzet, osmotic minipump at 0.30 μg/h) in 12–14 week old animals, followed by a 4‐week high salt ingestion (0.9% NaCl in drinking water; mice with UNX‐Aldo).

### 2.9 Western immunoblotting

Tissue samples were homogenized in radio‐immunoprecipitation assay (RIPA, R0278, Sigma) buffer mixed with 1% protease inhibitor cocktail (P8340, Sigma). Protein concentration was measured by Bradford assay. Equal amounts of protein were loaded for gel electrophoresis. After blotting, membranes (Hybond‐P, GE Healthcare) were probed with rabbit polyclonal anti‐ESAM antibody (1:1000, ab74777, Abcam), followed by incubation with HRP linked secondary antibodies (1:5000, 7074 S, Cell Signaling). Enhanced chemiluminescence was visualized autoradiographically by ChemiDoc XRS+ (Bio Rad). Protein expression was normalized for the total loaded protein using a BIO‐RAD TGX Stain‐Free FastCast Acrylamide Kit (Cat# 1610183), ChemiDoc imaging system, and Image Lab Software (Bio Rad).

### 2.10 Cytokine array analysis

Tissue samples were homogenized in radio‐immunoprecipitation assay (RIPA, R0278, Sigma) buffer mixed with 1% protease inhibitor cocktail (P8340, Sigma). Protein concentration was measured with Pierce BCA protein assay kit (23,227, ThermoScientific). Samples from experimental wild type and ESAM KO groups (*n* = 4 per group) were analyzed with a Proteome Profiler Mouse XL Cytokine Array (Cat# ARY028, R&D systems) according to the manufacturer's instructions. Quantification of the spot density was performed using Alpha View (ProteinSimple). Resulting figures were plotted in GraphPad Prism 9.2.

### 2.11 Statistical analysis

All statistical analyses were performed using GraphPad Prism Software. Data comparison between wild type and ESAM KO groups performed with unpaired *t*‐test with Welch's correction. Experiments involving four groups (WT and ESAM KO, UNX and UNXA) were analyzed with two‐way ANOVA followed by Tukey's post‐hoc test for multiple comparisons or with two‐tailed, unpaired Student's *t*‐test, as appropriate. Data are expressed as mean ± SEM. *p* < 0.05 was considered statistically significant.

## 3 RESULTS

### 3.1 ESAM ^−/−^ mice exhibit LV diastolic dysfunction

**FIGURE 1:**
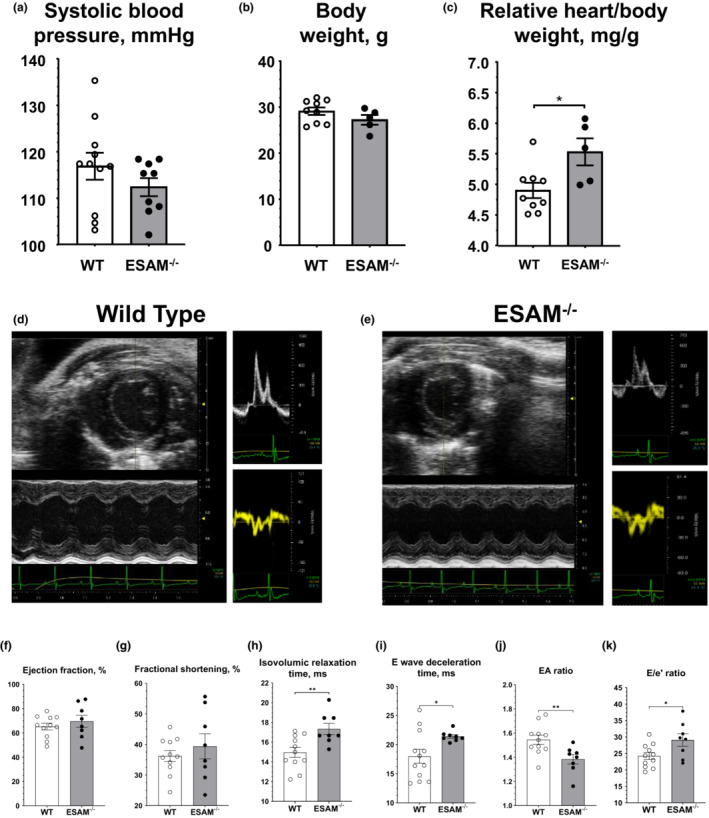
ESAM^−/−^ mice exhibit LV diastolic dysfunction. Systolic arterial blood pressure (a), body (b), and normalized heart weight (c), as well as representative echocardiogram images (d, e) and summary data (f–k) of echocardiogram parameters in ESAM^−/−^ and wild type (WT) mice (*N* = 9–11). Data are mean ± SEM, compared with unpaired *t*‐test with Welch's correction, *p < 0.05, **p < 0.01.

Sixteen‐ to twenty‐weeks old male ESAM^−/−^ mice had similar body weight and systolic arterial blood pressure, but exhibited increased normalized heart weights when compared to aged‐matched wild type controls (Figure 1a–c). Moreover, using echocardiography (Figure 1d,e), we found that the ejection fraction and fractional shortening, parameters representing LV systolic function were not significantly different between ESAM^−/−^ and wild type mice (Figure 1f,g). ESAM^−/−^ mice however exhibited LV diastolic dysfunction, as indicated by a significantly reduced E/A ratio (E = early, A = late mitral inflow peak velocities), increased E/e’ ratio, isovolumic relaxation time, and E wave deceleration time (Figure 1h–k).

### 3.2 Reduced myocardial vascular density and impaired angiogenesis in ESAM ^−/−^ mice

**FIGURE 2:**
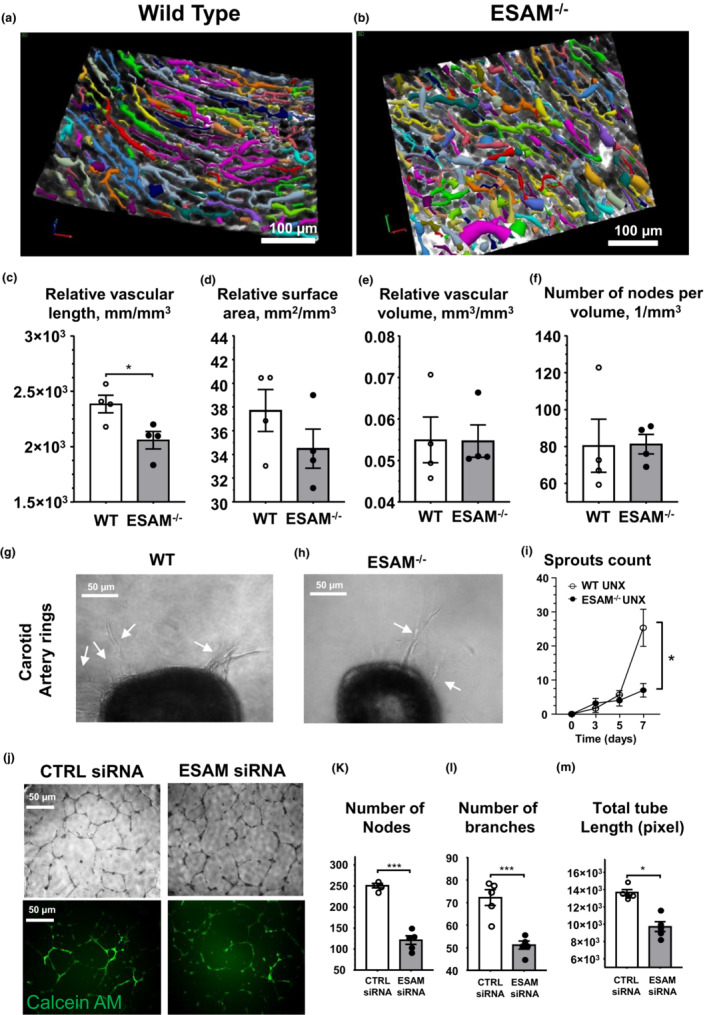
Vascular rarefaction in the heart of ESAM^−/−^ mice. Representative images of 3‐D reconstruction of blood vessels in the myocardium (a, b) and summary data (c–f) of vascular density and branching parameters in ESAM^−/−^ and wild type (WT) mice (*N* = 4–4). Representative images of collagen‐embedded carotid artery rings (g, h) and summary data (i) of the number of endothelial spurts during the 7‐day culture in ESAM^−/−^ and wild type (WT) mice (*N* = 4–4). Representative images of tube formation (j) and summary data (k–m) of tube length and branching parameters in HUVECs treated with scrambled (CTRL) and ESAM siRNA (data are from triplicate experiments). Data presented as mean ± SEM compared with unpaired *t*‐test with Welch's correction, *p < 0.05, **p < 0.01, ***p < 0.001. Sprouts assay data were analyzed using two‐way ANOVA with repeated measures, *p < 0.05.

To examine the impact of ESAM deletion on myocardial vascular density, we used an unbiased, automated tracing and 3D reconstruction approach applied to the coronary microcirculation. We found that ESAM^−/−^ mice have a significantly reduced total vascular length and we observed a trend toward a reduced microvascular surface area when compared to controls (Figure 2a–f).

To next investigate the angiogenic potential of arteries in ESAM^−/−^ mice, we employed ex vivo and in vitro angiogenic assays. We found that collagen embedded carotid arteries of ESAM^−/−^ mice displayed a significantly reduced number of endothelial sprouts growing during the 7‐day observation period (Figure 2g–i). Moreover, in cultured HUVECs we found that siRNA‐mediated ESAM knockdown significantly impaired endothelial tube formation, as indicated by a reduced number of nodes, branches, and total tube lengths (Figure 2j–m).

### 3.3 Increased myocardial neutrophils and changes in proinflammatory profile in ESAM ^−/−^ mice

**FIGURE 3:**
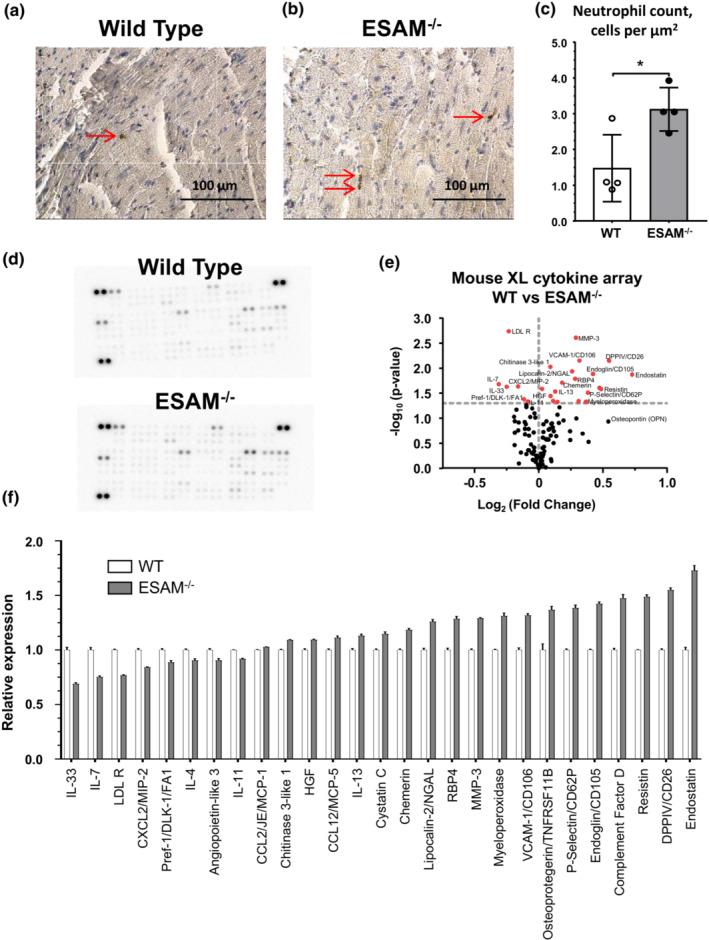
Neutrophil infiltration and pro‐inflammatory changes in the myocardium of ESAM^−/−^ mice. Representative images of immunohistochemical staining for myeloperoxidase (a, b) and summary data (c) of quantification of the number of myeloperoxidase positive cells in myocardial sections in ESAM^−/−^ and wild type (WT) mice (*N* = 4–4). Representative images of dot‐blot based cytokine array (d), volcano plot (e), and summary data (f) of differentially expressed inflammatory mediators in ESAM^−/−^ and wild type (WT) mice (*N* = 4–4). Data presented as mean ± SEM compared with unpaired *t*‐test with Welch's correction, *p < 0.05.

Heart sections were immuno‐labeled for myeloperoxidase to estimate cardiac neutrophil infiltration. We found an increased number of myeloperoxidase positive cells in ESAM^−/−^ mice when compared to controls (Figure 3a–c). These changes in the heart of ESAM^−/−^ mice were accompanied by changes in the pro‐inflammatory profile, including altered levels of inflammatory mediators, additional adhesion molecules, cytokines, and chemokines (Figure 3d–f).

### 3.4 Worsening of LV diastolic dysfunction after hemodynamic stress in ESAM ^−/−^ mice

**FIGURE 4:**
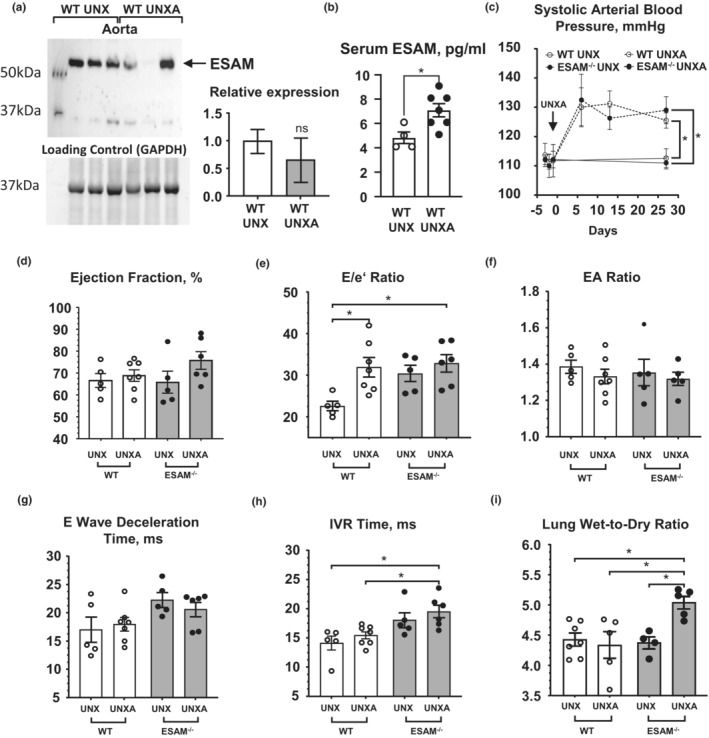
ESAM^−/−^ mice with UNX‐Aldo display worsening of LV diastolic dysfunction. Western immunoblot and summary data (*n* = 4 in each group) of ESAM expression in the aorta (a) and serum ESAM levels (b) in wild type (WT) mice with UNX‐Aldo (UNXA) procedures (*N* = 3–7). Summary data of systolic blood pressure changes (c) and summary data (d‐h) of echocardiogram parameters, as well as the calculated wet‐to‐dry ratio (i) in ESAM^−/−^ and wild type (WT) mice, with or without UNX‐Aldo procedures (*N* = 5–7). Data presented as mean ± SEM compared with unpaired *t*‐test with Welch's correction, *p < 0.05. A two‐way ANOVA with Tukey's multiple comparison test was used where appropriate, *p < 0.05.

We induced experimental volume and pressure overload in mice by performing uninephrectomy and aldosterone infusion (UNX‐Aldo) followed by 4‐week high salt ingestion. Interestingly, we found that the UNX‐Aldo procedure significantly elevated serum levels of soluble ESAM in wild type mice (Figure 4a,b). The UNX‐Aldo procedure in wild type mice induced LV diastolic dysfunction, without altering LV systolic parameters (Figure 4d–h). ESAM^−/−^ mice with UNX‐Aldo displayed similar increases in systolic blood pressure as WT controls (Figure 4c), whereas among the echocardiographic indices of LV diastolic dysfunction, IVRT declined further in ESAM^−/−^ mice with UNX‐Aldo (Figure 4d–h). Notably, ESAM^−/−^ mice with UNX‐Aldo also developed pulmonary edema, when compared to wild type mice with UNX‐Aldo (Figure 4i).

## 4 DISCUSSION

The salient findings of this study are that ESAM^−/−^ mice display a characteristic LV diastolic dysfunction, which is accompanied by reduced coronary vascular density and impaired angiogenesis. Cardiovascular phenotypic changes in the ESAM^−/−^ mice were also associated with increased myeloperoxidase‐positive neutrophil infiltration and altered inflammatory mediator and cytokine profiles in the myocardium. Furthermore, after experimental hemodynamic stress, ESAM^−/−^ mice displayed a worsening of LV diastolic function and developed significant pulmonary congestion. Thus, we propose that ESAM plays an important role in the maintenance of myocardial vascularization and LV diastolic function under basal and hemodynamic stress conditions.

The observed cardiovascular phenotypic changes in the ESAM^−/−^ mice were reminiscent to that seen in patients with heart failure with preserved ejection fraction (HFpEF, otherwise known as diastolic heart failure) (Oktay et al., 2013; Steinberg et al., 2012). Prior studies (Dryer et al., 2018), including our own observations (Davila et al., 2019; Dou et al., 2017), have shown that HFpEF patients often display a coronary perfusion deficit due to the dysfunctional microvascular endothelium (Crea et al., 2017; Paulus & Tschope, 2013; Yang et al., 2020), but the molecular interrelationships between coronary microvascular and LV diastolic dysfunction remain incompletely understood. Results from the present study indicate that ESAM deficiency is associated with the development of LV diastolic dysfunction in mice. Interestingly, we found that the serum level of soluble ESAM was elevated in the wild type UNX‐Aldo mice, an accepted mouse model of HFpEF (Valero‐Muñoz et al., 2016), displaying characteristic LV diastolic dysfunction. This observation aligns with available clinical data reporting increases in serum ESAM levels in patients with an enhanced risk for myocardial infarction, heart failure hospitalizations, and death (Park et al., 2015; Ren et al., 2017). Like many other adhesion molecules, the extracellular domain of ESAM can be excessively cleaved, and secreted into the bloodstream in a soluble form during various pathological conditions. Notable, in the Dallas Heart Study, involving 2442 participants without prior cardiovascular disease soluble ESAM, but not soluble ICAM‐1 or soluble VCAM‐1, levels were associated with incident atherosclerotic and total cardiovascular disease (Ren et al., 2017).

One possible mechanism through which ESAM deficiency and/or its excess cleavage could contribute to the development LV diastolic dysfunction can be attributed to its role in regulating microvascular density under stress conditions. LV diastolic dysfunction in HFpEF commonly arises from LV hypertrophy (Gazewood & Turner, 2017; Pfeffer et al., 2019); LV hypertrophy requires higher metabolic demands and signals for an increased coronary perfusion which is, in the long term, achieved by increasing the number of newly formed coronary blood vessels. Since ESAM was shown to play a role in the angiogenic process (Inoue et al., 2010), it is possible that an excessively cleaved or dysfunctional ESAM results in an impaired angiogenic response, and as cardiac hypertrophy progresses this could lead to LV diastolic dysfunction.

Data from this study moreover indicate that the reduced coronary microvascular density in ESAM^−/−^ mice hearts is accompanied by inflammatory cell infiltration and a pro‐inflammatory mediator shift. Microvascular dysfunction in HFpEF is commonly associated with elevated cardiac levels of markers of oxidative stress and pro‐inflammatory mediators (Cuijpers et al., 2020; Paulus & Tschope, 2013). It is debated whether pro‐inflammatory changes are the cause or the consequence of coronary microvascular dysfunction and LV diastolic dysfunction in HFpEF. Based on our results, we suggest that the reduced vascularization may cause absolute or relative myocardial perfusion deficits in the ESAM^−/−^ mice heart, which in turn drives pro‐inflammatory changes. Alternate possibilities as well as the mechanisms by which downregulated or dysfunctional ESAM promotes increased neutrophil infiltration and causes changes in the pro‐inflammatory profile have yet to be elucidated.

To the best of our knowledge, the current study is the first to describe that global ESAM deficiency in mice is accompanied by LV diastolic dysfunction. Results from this study suggest an impaired angiogenic response and subsequent pro‐inflammatory changes behind this phenomenon. Our study also implies that ESAM could maintain LV diastolic dysfunction during a hemodynamic challenge, and that soluble form of ESAM may have diagnostic implications in assessing the progression of LV diastolic dysfunction in HFpEF patients.

## AUTHOR CONTRIBUTIONS

Zsolt Bagi conceptualized the project; Zsolt Bagi, Vadym Buncha, Liwei Lang, and Katie Anne Fopiano performed the experiments. Vadym Buncha and Zsolt Bagi wrote the original draft of the manuscript. Vadym Buncha, Liwei Lang, Katie Anne Fopiano, Anatolij Horuzsko, Gaston Kapuku, and Zsolt Bagi reviewed and edited the manuscript. Zsolt Bagi supervised the research.

## FUNDING INFORMATION

This work was supported by awards from the American Heart Association [917057 to VB] and National Institutes of Health, National Institute on Aging [R01AG054651 to ZB].

## CONFLICT OF INTEREST STATEMENT

The authors declare no conflict of interests.
